# Genetic Optimization-Based Consensus Control of Multi-Agent 6-DoF UAV System

**DOI:** 10.3390/s20123576

**Published:** 2020-06-24

**Authors:** Aws Abdulsalam Najm, Ibraheem Kasim Ibraheem, Ahmad Taher Azar, Amjad J. Humaidi

**Affiliations:** 1 Department of electrical engineering, College of engineering, University of Baghdad, Baghdad 10001, Iraq; aws.najm@coeng.uobaghdad.edu.iq (A.A.N.); ibraheemki@coeng.uobaghdad.edu.iq (I.K.I.); 2 Robotics and Internet-of-Things Lab (RIOTU), Prince Sultan University, Riyadh 11586, Saudi Arabia; 3 Faculty of Computers and Artificial Intelligence, Benha University, Benha 13518, Egypt; 4 Department of Control and Systems Engineering, University of Technology, Baghdad 10001, Iraq; 601116@uotechnology.edu.iq

**Keywords:** hybrid control system, nonlinear PID, active disturbance rejection control, quadrotor system, unmanned aerial vehicle

## Abstract

A consensus control law is proposed for a multi-agent system of quadrotors with leader–follower communication topology for three quadrotor agents. The genetic algorithm (GA) is the proposed optimization technique to tune the consensus control parameters. The complete nonlinear model is used without any further simplifications in the simulations, while simplification in the model is used to theoretically design the controller. Different case studies and tests are done (i.e., trajectory tracking formation and switching topology) to show the effectiveness of the proposed controller. The results show good performance in all tests while achieving the consensus of the desired formations.

## 1. Introduction

In the last decade, unmanned aircrafts (UAs) have attracted researchers in different technical and scientific communities. UAs have a wide range of applications in various fields, such as agriculture, healthcare, and entertainment. Due to the great variety of UA types, UAs then can be classified by their application, range, altitude, endurance, vehicle type, and size. Many UA controlling missions are difficult, and some are impossible to accomplish using only a single agent UA, but instead a cooperative control must be designed for multiple agents to accomplish these missions. Interest in cooperative control of multi-agent systems (MASs) has recently increased, because of its broad applications, such as fire detection [1]. A literature survey [2] summarized some of the potential applications of the multi-agent system, such as large object transportation, which was done in the GRASP laboratory at the University of Pennsylvania, surveillance and searching of objects, quadrotor localization in outdoor environment, which was done at the Czech Technical University, and object transportation missions [3]. There are many cooperation control problems, such as consensus control, formation control, flocking control, and so on. Another important research field is optimization, which has attracted many researchers due to the advantages provided by optimization, especially with complex and nonlinear systems. There has been much research in this field with different optimization techniques, such as genetic algorithm (GA) optimization [4,5], particle swarm optimization (PSO) [6], ant colony optimization (ACO) [7], as well as hybridization of two or more techniques [8,9].

### 1.1. Multi-Agent Systems

Generally, MASs are systems composed of multiple cooperating agents. Two significant abilities exist in agents that are controlled by computers (computerized agents). Firstly, they have the ability to do an independent task (i.e., deciding what to do to fulfill their task goals). Secondly, they have the ability to communicate with each other (i.e., they not only swap data, but they also participate with their peers by negotiation, cooperation, coordination, and so on) [10]. As mentioned earlier, there are many cooperative control problems. In this paper, light will be shed on the most common cooperating control problems, i.e., consensus control and formation control.

#### 1.1.1. Consensus Control

**Figure 1:**
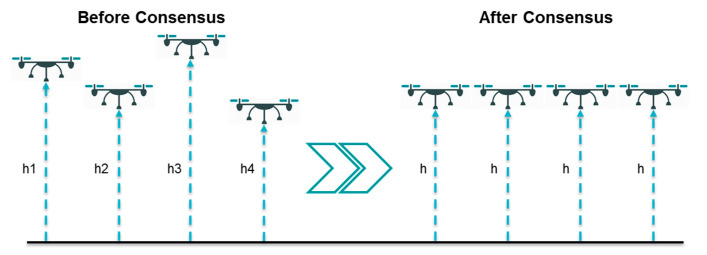
Consensus problem.

Consensus problem is closely related to formation control (forming a specific geometrical shape, which is discussed next), flocking (a behavior presented when a group of birds are foraging or in-flight), and distributed estimation [11]; thus, it is an important and essential problem in the field of cooperative control. Consensus means reaching an agreement between multiple agents to achieve a common final state by communicating with each other through a communication network [12]. Figure 1 shows the consensus problem for four agents in their elevations only, where the four agents on the left side have different elevations above the ground (i.e., $h_{1}$, $h_{2}$, $h_{3}$, and $h_{4}$) before applying the consensus control algorithm, while the agents on the right hand side present the agents after applying the consensus controller and reaching consensus in the elevation (i.e., h).

#### 1.1.2. Formation Control

**Figure 2:**
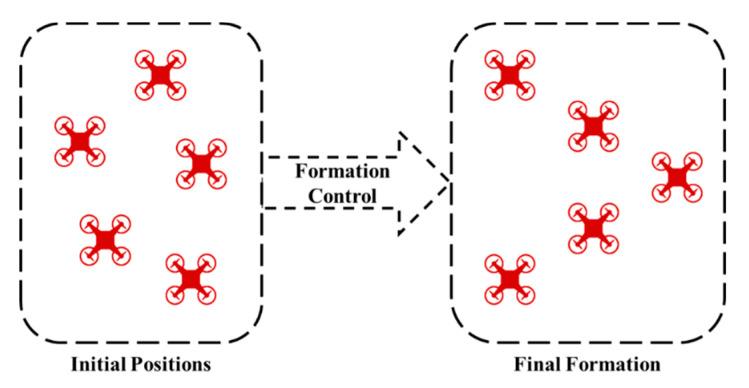
Before and after applying formation control on 5 agents.

The formation control problem requires that all the agents reach a specific geometrical formation; thus, formation control differs from consensus by adding a distance to achieve the geometrical shape by all the agents; therefore, the formation control can be varied into consensus control by making the additional distance equal to zero. There are two cases for the formation control problem according to information known to agents. The first case is where the desired formation and its location are known for the team agents (i.e., centralized case). The second case is where the desired formation is known for the agents, while its location must be achieved by the team agents after negotiation [13]. Figure 2 shows five agents before and after applying formation control. In the initial positions, the agents are randomly distributed in two dimensions, and the heights of the agents are not taken into consideration. A formation controller is applied to achieve a triangle geometry shape, which is presented in the final formation.

Consensus and formation control algorithms can be categorized into leaderless and leader–follower depending on how the agents are connected in the communication network represented using graph theory [14].

#### 1.1.3. Optimization Techniques

Finding the optimum performance index by tuning the controller parameters without any constraint is the purpose of the optimization techniques in this paper; therefore, the optimization technique used must be unconstrained. There are several methods to solve an unconstrained minimization problem, which can be classified as nongradient methods (e.g., random search method, grid search method, simplex method, etc.) and gradient methods (e.g., Fletcher–Reeves method, Newton’s method, Marquardt method, etc.).

For simple problems that involve a small number of variables, the nongradient methods are efficient, while the gradient methods require the first and sometimes the second derivative of the objective function (performance index). Both methods are not efficient in the case of this work due to the number of variables available in the tuning process and the complexity gained by getting the first or second derivative of the performance index used. Therefore, the modern optimization techniques are the best choice for the tuning and optimization problem of the proposed controller.

There is a wide range of modern methods available, such as genetic algorithms, simulated annealing, PSO, ACO, etc., which can be used in the tuning process. Due to the ability of the GA to avoid local optimal solutions, it was chosen as the optimization method to tune the parameters of the proposed controller.

#### 1.1.4. Genetic Algorithm

The genetic algorithm (GA) is categorized as an evolutionary algorithm that is suitable in searching for a global solution. Inspiration in GA development comes from natural genetics working principles. Three main operators are used at each step to produce the next generation from the current one. These three operators are selection, crossover, and mutation, which are briefly explained in the following [4]:

(1) Selection: The selection operation forms a mating pool for the next generation by selecting genes (parents) from the existing generation.
(2) Crossover: The crossover process combines two of the parents to form the next generation (children).
(3) Mutation: The mutation forms new children by applying random changes to the parents.

**Figure 3:**
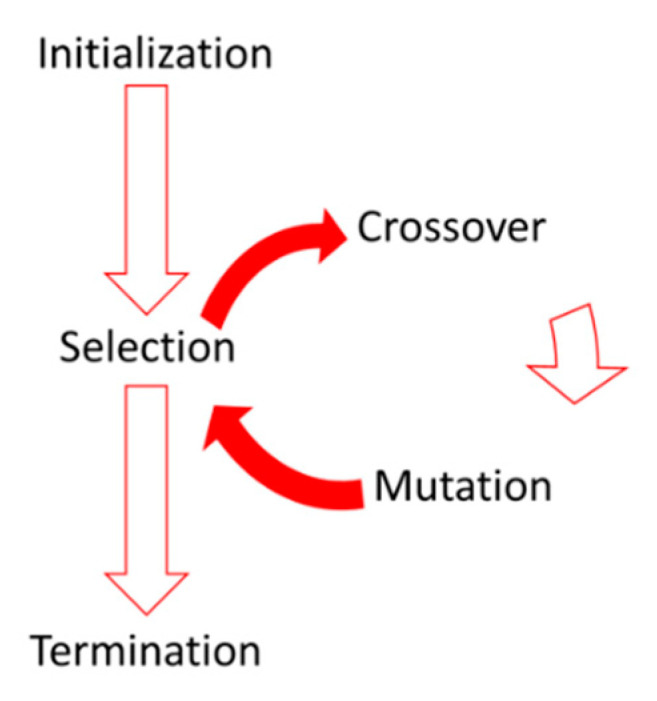
Steps diagram of the genetic algorithm (GA).

A concise diagram for the GA steps is shown in Figure 3. In this paper, the MATLAB function ga was used in the optimization and tuning processes.

### 1.2. Literature Survey

There is a large variation of research on cooperative control techniques for MASs. Getting an agreement between the agents (i.e., consensus) was the aim of some of the researchers, while others intended to obtain a specific formation for the MAS under some constraints (i.e., communication topology, leader/leaderless, …, etc.). This depends on the application where the control technique is used. Some of the recent research, such as authors in [15], proposed a distributed adaptive controller for a class of an under-actuated Lagrangian system to track a dynamic leader and control the actuated variables, while being subjected to parameter uncertainties and external disturbance with or without actuator faults. In [16], the authors studied the distributed consensus problem for a team of quadrotors with fixed and switching topologies after transforming the quadrotor model to a simplified one using feedback linearization technique. Furthermore, others in [17] presented a two-layer hierarchical model predictive control (MPC) to stabilize a quadrotors team subjected to some constraints. Authors in [18] designed an adaptive consensus controller based on multiple surface control (MSC) to achieve a specific leader–follower formation for a team of quadrotors, taking into consideration the uncertainties generated by the ground effects. The results presented showed good performance with good adaptation of the slack variables and the disturbance of landing or taking off. Five different controllers were developed in [19]. Two of them (i.e., the nonlinear suboptimal $H_{\infty }$ control technique and the integral backstepping (IBS) controller) were based on Lyapunov theory and were proposed for trajectory tracking and leader–follower formation control. The others were proportional derivative square ($PD^{2}$), which was compared with $H_{\infty }$ control by applying them on the attitude system and the linear quadratic regulator (LQR) with the optimal iterative LQR, which were applied and compared for attitude, trajectory tracking, and formation control. Researchers in [20] used a simplified quadrotor model to develop a novel distributed consensus algorithm, while one of the uniform constant delays and asynchronous time-varying delays existed as a communication delay. A backstepping based on proportional integral derivative (PID) control was designed in [21] to solve the formation problem for a team of quadrotors in a decentralized manner. The system stability and retention of the desired geometric formation with or without the existence of the obstacles were presented in the simulations. Authors in [22] proposed a novel consensus leader–follower formation control based on a non-smooth backstepping design. The different numerical simulations verified the effectiveness of the proposed controller, and theoretical analysis showed that all the quadrotors converged to the desired formation.

### 1.3. Motivation and Contributions

In the literature survey mentioned previously, a simplified model for the UA system was used in most of the research. This issue and others motivated us to simulate the complete UA quadrotor system model by using a proposed consensus controller based on optimization techniques. Moreover, the leader–follower topology was used in some case studies, where the leader agent was designed as a complete quadrotor system controlled by the Nonlinear PID (NLPID) and Improved Active Disturbance Rejection Control (IADRC) scheme proposed in our previous work, with the followers controlled by the controller scheme proposed in this work. 

The main contributions of this paper can be summarized as follows:

- Proposing a new consensus controlling scheme based on optimization techniques for a MAS of 6-degree of freedom (DOF) quadrotors.
- Simulating a leader–follower topology in some cases by adding additional terms to control the geometry of the agents, and if these terms are equalized to zero, the agents goes to the consensus case.

### 1.4. Notations

In this paper, vectors and matrices are denoted by bold symbols (e.g., A or a). Dot notation is used for time derivative. In this notation a dot is placed above the function name to denote the time derivative of a function (e.g., the first and second derivatives of x with respect to time t are denoted by $\dot{x}$, $\ddot{x}$). The integration in this paper is denoted by the symbol ∫ (e.g., the integration of the function x with respect to time t between a lower limit a and upper limit b is denoted by $\int_{a}^{b}xdt$). The sign ⊗ is used in this paper as a Kronecker product operation.

### 1.5. Paper Organization

The rest of the paper is organized as follows: Some preliminaries on the quadrotor mathematical model, graph theory, mathematical representation of consensus control, and finally the problem statement of this paper are given in Section 2; thereafter, the proposed consensus control scheme is demonstrated in Section 3. Next, some simulations and tests are performed and discussed in Section 4. Finally, Section 5 includes the paper conclusions and some of the future aspects.

## 2. Preliminaries and Problem Statement

### 2.1. Quadrotor Mathematical Model

As mentioned in our previous works, the mathematical model of the quadrotor system is derived in Appendix A based on Newton–Euler equation and can be represented in Equation (1), while Newton’s law is used to obtain Equation (2), which is helpful to design a controller for the under-actuated quadrotor system using the acceleration in 3 dimensions [23,24]; all the parameters used are described in Table 1.

(1) $$\{\begin{array}{ll} \left\{ \begin{array}{l} \dot{x}=c\left(\psi \right)c\left(\theta \right)u+\left[c\left(\psi \right)s\left(\phi \right)s\left(\theta \right)-c\left(\phi \right)s\left(\psi \right)\right]v+\left[s\left(\phi \right)s\left(\psi \right)+c\left(\phi \right)c\left(\psi \right)s\left(\theta \right)\right]w\\ \dot{u}=rv-qw-gs\left(\theta \right)+\frac{f_{wx}}{m} \end{array}\right. & \left(a\right)\\ \left\{ \begin{array}{l} \dot{y}=c\left(\theta \right)s\left(\psi \right)u+\left[c\left(\phi \right)c\left(\psi \right)+s\left(\phi \right)s\left(\psi \right)s\left(\theta \right)\right]v+\left[c\left(\phi \right)s\left(\psi \right)s\left(\theta \right)-c\left(\psi \right)s\left(\phi \right)\right]w\\ \dot{v}=-ru+pw+gs\left(\phi \right)c\left(\theta \right)+\frac{f_{wy}}{m} \end{array}\right. & \left(b\right)\\ \left\{ \begin{array}{l} \dot{z}=-s\left(\theta \right)u+c\left(\theta \right)s\left(\phi \right)v+c\left(\phi \right)c\left(\theta \right)w\\ \dot{w}=qu-pv+gc\left(\theta \right)c\left(\phi \right)+\frac{f_{wz}-f_{t}}{m} \end{array}\right. & \left(c\right)\\ \left\{ \begin{array}{l} \dot{\phi }=p+s\left(\phi \right)t\left(\theta \right)q+c\left(\phi \right)t\left(\theta \right)r\\ \dot{p}=\frac{I_{yy}-I_{zz}}{I_{xx}}rq+\frac{\tau_{x}+\tau_{wx}}{I_{xx}} \end{array}\right. & \left(d\right)\\ \left\{ \begin{array}{l} \dot{\theta }=c\left(\phi \right)q-s\left(\phi \right)r\\ \dot{q}=\frac{I_{zz}-I_{xx}}{I_{yy}}pr+\frac{\tau_{y}+\tau_{wy}}{I_{yy}} \end{array}\right. & \left(e\right)\\ \left\{ \begin{array}{l} \dot{\psi }=\frac{s\left(\phi \right)}{c\left(\theta \right)}q+\frac{c\left(\phi \right)}{c\left(\theta \right)}r\\ \dot{r}=\frac{I_{xx}-I_{yy}}{I_{zz}}pq+\frac{\tau_{z}+\tau_{wz}}{I_{zz}} \end{array}\right. & \left(f\right) \end{array}$$

(2) $$\{\begin{array}{l} \ddot{x}=-\frac{f_{t}}{m}\left[s\left(\phi \right)s\left(\psi \right)+c\left(\phi \right)c\left(\psi \right)s\left(\theta \right)\right]\\ \ddot{y}=-\frac{f_{t}}{m}\left[c\left(\phi \right)s\left(\psi \right)s\left(\theta \right)-c\left(\psi \right)s\left(\phi \right)\right]\\ \ddot{z}=-\frac{f_{t}}{m}\left[c\left(\phi \right)c\left(\theta \right)\right]+g \end{array}$$

### 2.2. Graph Theory

The mathematical preliminaries of the consensus control are important to be studied and understood to pave the way for dealing with the consensus control. The consensus control design procedure consists of a number of consecutive steps, which are listed below:

- Determining the number of agents.
- Deriving a graph based on agent communication.
- Deriving an adjacency matrix from the graph.
- Converting the adjacency matrix into a Laplacian matrix.
- Using the Laplacian matrix to design the consensus controller.

Thus, graph theory plays a key role in these steps, since it can reflect the communication topology of MASs [25].

Graph theory is commonly used in consensus control to describe the topology of the connection between the agents. Connection topology can be categorized as directed and undirected based on the information transmitted between the agents.

A graph G is built upon a finite set, which has a limited number of elements. This finite set is called vertex set V, which includes the vertices of the graph. Assuming a graph G has n vertices, then the vertex set V can be represented as $V=\left\{V_{1},V_{2},\ldots ,V_{n}\right\}$. A set of two vertices from V in the form $E=\left\{V_{i},V_{j}\right\}\subset V\times V$, where i,j=1,2,…,n and i≠j, is called edge, while a set of edges is denoted by E. Sometimes another notation is used for the vertices and edges of the graph G as V(G) and E(G), respectively, and for simplicity edge $\left\{V_{i},V_{j}\right\}$ is denoted as $V_{i}V_{j}$.

The directed graph (digraph) is defined as a graph D that consists of a vertex set V(D) and directed edge set E(D). In a directed graph, the direction of the edge is shown by an arrow. The graph is undefined if the vertex set is undefined.

#### 2.2.1. Adjacency and Degree Matrices

For an undirected graph G, each vertex $V_{i}$ has a degree $d\left(V_{i}\right)$. This degree is equal to the number of vertices adjacent to $V_{i}$ in G. All the vertices’ degrees of the graph are represented in matrix form as a diagonal matrix, which is called a degree matrix and is given as

(3) $$\Delta \left(G\right)=\left[\begin{array}{cccc} d\left(V_{1}\right) & 0 & \cdots & 0\\ 0 & d\left(V_{2}\right) & \cdots & 0\\ \vdots & \vdots & \ddots & \vdots \\ 0 & 0 & \cdots & d\left(V_{n}\right) \end{array}\right]$$

where Δ( ) is the degree matrix of graph G, and n is the number of vertices or agents.

The adjacency matrix A(G) is a symmetrical matrix that reflects adjacency links between the vertices in graph G, and can be derived from the following relationship:

(4) $${\left[A\left(G\right)\right]}_{ij}=\{\begin{array}{ll} 1 & \mathrm{for}\;V_{i}V_{j}\in E\left(G\right)\\ 0 & \mathrm{otherwise} \end{array}$$

In all previously mentioned cases, the edge weights are considered to be one; but sometimes other weights are used instead of one. Generally, for weighted digraphs, the adjacency matrix is given as

(5) $${\left[A\left(D\right)\right]}_{ij}=\{\begin{array}{ll} {𝓌}_{ij} & \mathrm{for}\;\left(V_{j},V_{i}\right)\in E\left(D\right)\\ 0 & \mathrm{otherwise} \end{array}$$

where ${𝓌}_{ij}$ is the weight for the edge between vertices $V_{i}\;\mathrm{and}\;V_{j}$. The diagonal degree matrix is given as

(6) $$\Delta \left(D\right)=\left[\begin{array}{cccc} d_{in}\left(V_{1}\right) & 0 & \cdots & 0\\ 0 & d_{in}\left(V_{2}\right) & \cdots & 0\\ \vdots & \vdots & \ddots & \vdots \\ 0 & 0 & \cdots & d_{in}\left(V_{n}\right) \end{array}\right]$$

where $d_{in}\left(V_{i}\right)$ is the weighted in-degree of vertex $V_{i}$, which represents the sum of the weights of the edge that is coming from vertex $V_{j}$ into vertex $V_{i}$ and is given in the following relation:

(7) $$d_{in}\left(V_{i}\right)=\underset{\left\{j|\left(V_{j},V_{i}\right)\in E\left(D\right)\right\}}{\sum }{𝓌}_{ij}$$

#### 2.2.2. Laplacian Matrix

There are many ways to represent the Laplacian of a graph. The most straight forward method is given as follows:

(8) L(G)=Δ(G)−A(G)

where Δ(G) is the degree matrix of graph G, and A(G) is the adjacency matrix for graph G. 

### 2.3. Mathematical Representation of Consensus Control

Consensus control stability and convergence analysis is a complex method due to the fact that consensus control is dealing with MAS, not a single agent. This is the reason why researchers in their publications simplify the system modeling to simplify the analysis approach, unlike the system modeling in a single agent. Some of the commonly used approaches for consensus control are the single-integrator system, double-integrator, and higher-order MASs, which are described next [25].

#### 2.3.1. Single-Integrator MAS

MASs of single-integrator dynamics can be mathematically represented as follows:

(9) $${\dot{x}}_{i}=u_{i},\;i=1,2,\ldots ,N$$

where $x_{i}\in R^{n}$ are the states vector for agent i, $u_{i}\in R^{m}$ is the input vector for agent i, and N refers to the number of agents in the MAS. The consensus control is represented by

(10) $$u_{i}=K\overset{N}{\underset{j=1}{\sum }}\ell_{ij}\left(x_{i}-x_{j}\right)$$

where $\ell_{ij}$ is the (i,j) element of the Laplacian matrix, and $K\in R^{m\times n}$ is the control matrix, which needs to be determined.

To write the consensus in a general matrix form, substitute Equation (10) in Equation (9).

(11) $$\dot{X}=-KL\left(G\right)X$$

where $X={\left[x_{1}\;x_{2}\ldots x_{N}\right]}^{T}$, and L(G) is defined in Equation (8).

#### 2.3.2. Double-Integrator MAS

MASs of double-integrator dynamics can be mathematically represented as

(12) $$\{\begin{array}{l} {\dot{x}}_{i}=v_{i}\\ {\dot{v}}_{i}=u_{i} \end{array},\;i=1,2,\ldots ,N$$

where $x_{i},v_{i}\in R^{n}$ are the position and velocity vectors, respectively, for agent i, and $u_{i}\in R^{m}$ is the input vector for agent i. The consensus control is represented by

(13) $$u_{i}=K\overset{N}{\underset{j=1}{\sum }}\ell_{ij}\left(\left(x_{i}-x_{j}\right)+\gamma \left(v_{i}-v_{j}\right)\right)$$

The general matrix representation for the double-integrator MAS can be achieved by using Equation (12) with the controller in Equation (13) as follows:

(14) $$\left[\begin{array}{c} \dot{X}\\ \dot{V} \end{array}\right]=\left[\begin{array}{cc} 0_{n\times n} & I_{n}\\ -KL\left(G\right) & -\gamma KL\left(G\right) \end{array}\right]\left[\begin{array}{c} X\\ V \end{array}\right]$$

where $X={\left[x_{1}\;x_{2}\ldots x_{N}\right]}^{T}$ and $V={\left[v_{1}\;v_{2}\ldots v_{N}\right]}^{T}$.

#### 2.3.3. Higher-Order MAS

An extension for the single and double-integrator MASs is given as follows:

(15) $$\{\begin{array}{l} {\dot{x}}_{i}=Ax_{i}+Bu_{i}\\ y_{i}=Cx_{i} \end{array},i=1,2,\ldots ,N$$

where $A\in R^{n\times n},\;B\in R^{n\times m},\;C\in R^{p\times n},\;x_{i}\in R^{n}$ is the state vector for agent i, $u_{i}\in R^{m}$ is the input vector for agent i, and $y_{i}\in R^{p}$ is the output vector for agent i. The consensus control can be achieved using the control defined in Equation (10) applied on the MAS of Equation (15). Then, the matrix form representation is as follows:

(16) $$\dot{X}=\left(I_{N}⊗A-L\left(G\right)⊗BK\right)X$$

### 2.4. Problem Statement

A network of homogenous connected MAS is considered as a leader–follower connection, where each agent mathematical model can be represented in the compact form. The control signal of the leader is treated as a single agent control problem, while the followers’ controllers have to be designed and tuned based on the GA optimization technique such that they stabilize the unstable followers, reach consensus between all the MAS agents, and achieve desired formations while tracking different trajectories.

## 3. Proposed Controlling Scheme Design

**Figure 4:**
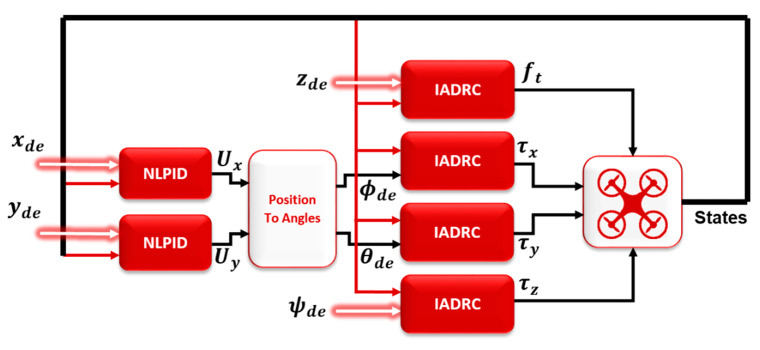
IADRC control for ILC and NLPID control for OLC of quadrotor system.

**Figure 5:**
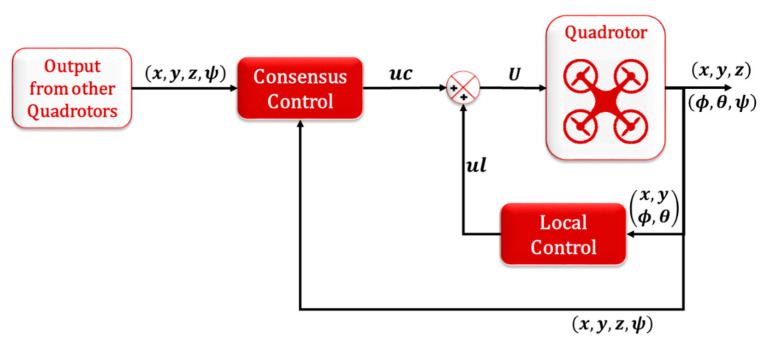
Local and consensus controllers.

The consensus control is proposed in this paper to achieve tracking formation for the MAS of the quadrotors while using the leader–follower connection topology between the agents (directed graph). The aim of the tracking formation is to keep the agents in a formation on the 3D space (x, y, z) and the angle of rotation around z-axis (ψ) while tracking the leader. Therefore, the leader control is different from the followers, which can be represented by normal quadrotor control using a combination of NLPID for the outer loop control (OLC) and IADRC for the inner loop control (ILC) of the quadrotor system, which was designed in our previous works [26,27,28] and is shown in Figure 4. The followers’ control is a mix of two controllers; the first is the local one and the second is the consensus controller, as shown in Figure 5.

The follower agent control system for (ϕ, θ) states are the IADRC designed for the leader agent, before proceeding in the proposed controller for the states (x, y, z, ψ) a brief explanation and equations for the NLPID and the IADRC are given.

### 3.1. Nonlinear PID (NLPID) Controller

The NLPID controller is developed based on the PID controller to be better while dealing with highly complicated nonlinear systems such as the 6-DOF quadrotor system. The NLPID controller is given in Equation (17).

(17) $$\{\begin{array}{l} U_{NLPID}=f_{1}\left(e\right)+f_{2}\left(\dot{e}\right)+f_{3}\left(\int e\;dt\right)\\ f_{i}\left(\varepsilon \right)=k_{i}\left(\varepsilon \right){\left|\varepsilon \right|}^{\alpha_{i}}sign\left(\varepsilon \right)\\ k_{i}\left(\varepsilon \right)=k_{i1}+\frac{k_{i2}}{1+exp\left(\mu_{i}\varepsilon^{2}\right)}\;i\in \left\{1,2,3\right\} \end{array}$$

where ε could be $e,\dot{e},$ or ∫e dt, $\alpha_{i}\in R^{+}$ and the function $k_{i}\left(\varepsilon \right)$ is a positive function with coefficients $k_{i1},k_{i2},\mu_{i}\in R^{+}$. The term $k_{i}\left(\varepsilon \right)$ is introduced here to make the NLPID controller more sensitive for small errors. Small errors which is approximate to zero, the value of the term $k_{i}\left(\varepsilon \right)$ approaches to the upper bound $k_{i1}+\frac{k_{i2}}{2}$, while for large errors, the term $k_{i}\left(\varepsilon \right)$ approaches the lower bound $k_{i1}$, this means that the limits of the term $k_{i}\left(\varepsilon \right)$ is bounded in the range $\left[k_{i1},k_{i1}+\frac{k_{i2}}{2}\right]$.

### 3.2. Improved Active Disturbance Rejection Control (IADRC)

ADRC, is in general, a grouping of three dynamic systems: Extended State Observer (ESO), Tracking Differentiator (TD), and State Error Feedback (SEF) controller. In our previous paper, IADRC is proposed, which is a grouping of the abovementioned three dynamic systems taking into consideration that linear ESO (LESO) is used, TD is replaced by improved TD (ITD) and that SEF is changed by NLPID control.

#### 3.2.1. Design of Linear Extended State Observer (LESO)

The key idea of this element is to convert the ESO with the error equation of the system to a disturbed asymptotical stable system, in which the high-gain eliminates the effect of the total disturbance error. The mathematical representation for the LESO used is given as,

(18) $$\left\{ \begin{array}{l} {\dot{z}}_{1}=z_{2}+\beta_{1}e_{1}\\ {\dot{z}}_{2}=z_{3}+\beta_{2}e_{1}\\ \;\vdots \\ {\dot{z}}_{\rho }=z_{\rho +1}+\beta_{\rho }e_{1}+b_{0}U\\ {\dot{z}}_{\rho +1}=\beta_{\rho +1}e_{1} \end{array}\right.$$

(19) $$U=u_{0}-\frac{z_{\rho +1}}{b_{0}}$$

where $u_{0}$ is control input, $e_{1}=\left(y-z_{1}\right)$, and $\beta_{i}$ are gain constants of the observer needed to be tuned, i∈{1,2,…,ρ,ρ+1}. $\beta_{i}$ = $c_{i}\omega_{0}$, $c_{i},i\in \left\{1,2,\ldots ,\rho ,\rho +1\right\}$ are relevant constants, and $\omega_{0}$ is the bandwidth of the observer and need to be chosen to get better estimation for the state and the disturbance with minimum OPI, and $z_{1},z_{2},\ldots ,z_{\rho +1}$ represent the states of the plant estimated by the ESO.

#### 3.2.2. Design of Improved Tracking Differentiator (ITD)

The aim of this element is to generate a smooth approximation of the reference signal and its derivatives for the control system, which can be mathematically represented as:

(20) $$\left\{ \begin{array}{l} {\dot{r}}_{1}=r_{2},\;r_{1}\left(0\right)=r_{10}\\ {\dot{r}}_{2}=r_{3},\;r_{2}\left(0\right)=r_{20}\\ \;\vdots \\ {\dot{r}}_{\rho }=-R^{\rho }tanh\left(\frac{\beta r_{1}-\left(1-\alpha \right)r}{\gamma }\right)-R^{\rho -1}r_{2}\left(t\right)-\ldots -Rr_{\rho }\left(t\right),\\ r_{\rho }\left(0\right)=r_{\rho 0} \end{array}\right.$$

where $\left\{r_{1},r_{2},\ldots ,r_{\rho }\right\}$ are the tracking signals (i.e., the reference signal and the derivatives of r) tracked by the differentiator signals and having initial values $\left\{r_{10},r_{20},\ldots ,r_{\rho 0}\right\}$. The parameters R,β,α and γ are the ITD parameters which have to be tuned to get the best tracking with small errors.

#### 3.2.3. Design of Nonlinear PD Controller (NLPD)

The NLPID will be used as the SEF in the IADRC, which is the same in Equation (17), but for general chain of integrators system of order ρ. The general chain of integrators system can be mathematically represented as:

(21) $$\left\{ \begin{array}{l} u_{0}=f_{1}\left(e\right)+f_{2}\left(\dot{e}\right)+\cdots +f_{\rho }\;\left(e^{\rho }\;\right)+f_{\rho +1}\left(\int e\;dt\right)\\ f_{i}\left(\varepsilon \right)=k_{i}\left(\varepsilon \right){\left|\varepsilon \right|}^{\alpha_{i}}sign\left(\varepsilon \right)\\ k_{i}\left(\varepsilon \right)=k_{i1}+\frac{k_{i2}}{1+exp\left(\mu_{i}\varepsilon^{2}\right)},\;i\in \left\{1,2,\ldots ,\rho +1\right\} \end{array}\right.$$

where ε could be $e,\dot{e},\ldots ,e^{\rho }\;or\;\int e\;dt,$ and $\left(k_{i1},k_{i2},\mu_{i},\alpha_{i}\right)$ are the controller parameters and are tuned to get minimum OPI. Due to the derivation in [24] of the IADRC, which changes the system into a chain of integrators, the integrator term in the NLPID will be neglected. The NLPD controller for general chain of integrators system of order ρ is represented as:

(22) $$\left\{ \begin{array}{l} u_{0}=f_{1}\left(e\right)+f_{2}\left(\dot{e}\right)+\cdots +f_{\rho }\left(e^{\rho }\right)\\ f_{i}\left(\varepsilon \right)=k_{i}\left(\varepsilon \right){\left|\varepsilon \right|}^{\alpha_{i}}sign\left(\varepsilon \right)\\ k_{i}\left(\varepsilon \right)=k_{i1}+\frac{k_{i2}}{1+exp\left(\mu_{i}\varepsilon^{2}\right)},\;i\in \left\{1,2,\ldots ,\rho \right\} \end{array}\right.$$

### 3.3. Consensus Control for x−y Position Subsystems

The x−y position dynamics of the follower agents are given by

(23) $$\{\begin{array}{l} {\ddot{x}}_{i}=U_{x}{}_{i}\\ {\ddot{y}}_{i}=U_{y}{}_{i} \end{array},\;i=1,2,\ldots ,N$$

where N is the number of agents, while the leader agent is denoted by 0. The proposed controller for x−y position subsystems can be represented as

(24) $$\{\begin{array}{l} U_{x}{}_{i}=ul_{x}{}_{i}+uc_{x}{}_{i}\\ U_{y}{}_{i}=ul_{y}{}_{i}+uc_{y}{}_{i} \end{array}$$

where ul and uc are the local and the consensus control signals for each agent, respectively. The consensus controller is designed as a single integrator system consensus controller, which is given by Equation (10) adding the formation position terms, while the local controller is designed to ensure that

$$\underset{t\to \infty }{\lim}\dot{x}=\underset{t\to \infty }{\lim}\dot{y}=0$$

and is given by

(25) $$\{\begin{array}{l} ul_{x}{}_{i}=-k_{x}{\dot{x}}_{i}\\ ul_{y}{}_{i}=-k_{y}{\dot{y}}_{i} \end{array}$$

where $k_{x}$ and $k_{y}$ are the controllers’ parameters, which need to be tuned and optimized to give better tracking performance. Then Equation (24) can be written as

(26) $$\{\begin{array}{l} U_{x}{}_{i}=-k_{x}{\dot{x}}_{i}+K_{x}\overset{N}{\underset{j=1}{\sum }}\ell_{ij}\left(\left(x_{i}-x_{j}-\varrho_{xi}\right)\right)\\ U_{y}{}_{i}=-k_{y}{\dot{y}}_{i}+K_{y}\overset{N}{\underset{j=1}{\sum }}\ell_{ij}\left(\left(y_{i}-y_{j}-\varrho_{yi}\right)\right) \end{array}$$

where $\varrho_{xi}$ and $\varrho_{yi}$ are the reference state in the x and y, respectively, and $K_{x}$ and $K_{y}\in R$ are the consensus control parameters, which need to be tuned and optimized to give better performance.

### 3.4. Consensus Control for z and ψ Subsystems

The dynamics of z subsystem is given as

(27) $$\{\begin{array}{l} \;\dot{z}=-s\left(\theta \right)u+c\left(\theta \right)s\left(\phi \right)v+c\left(\phi \right)c\left(\theta \right)w\\ \ddot{z}=\dot{w}=-\frac{f_{t}}{m}\left[c\left(\phi \right)c\left(\theta \right)\right]+g \end{array}$$

It is very clear that it is coupled and nonlinear. Therefore, to overcome this problem and design the consensus controller, the quadrotor system is assumed to be near the hovering point where ϕ=θ≈0. Then the dynamics of z subsystem is represented as follows:

(28) $$\{\begin{array}{l} \dot{z}=w\\ \dot{w}=-\frac{f_{t}}{m}+g \end{array}$$

which is a double integrator system and can be controlled using the consensus controller expressed in Equation (13), assuming that γ=1 and adding the formation position terms. The controller of the z subsystem is given as

(29) $$U_{z}{}_{i}=f_{t}=\overset{N}{\underset{j=1}{\sum }}\ell_{ij}\left(K_{z1}\left(z_{i}-z_{j}-\varrho_{zi}\right)+K_{z2}\left(w_{i}-w_{j}\right)\right)$$

while for the dynamics of ψ subsystem is given as

(30) $$\{\begin{array}{l} \dot{\psi }=\frac{s\left(\phi \right)}{c\left(\theta \right)}q+\frac{c\left(\phi \right)}{c\left(\theta \right)}r\\ \dot{r}=\frac{I_{xx}-I_{yy}}{I_{zz}}pq+\frac{\tau_{z}+\tau_{wz}}{I_{zz}} \end{array}$$

**Figure 6:**
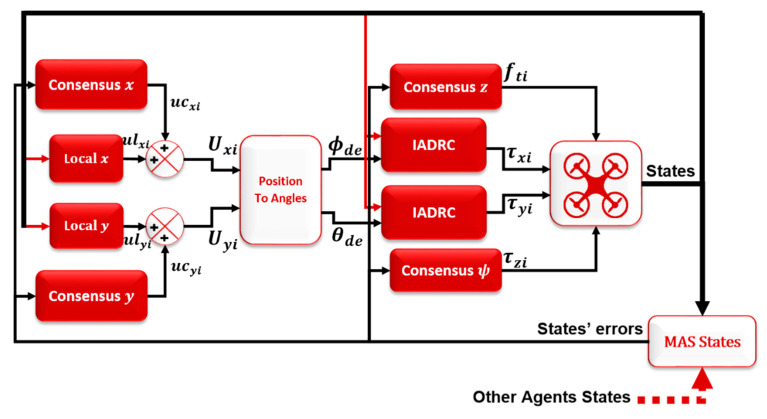
The overall ith quadrotor system with the consensus controller.

The assumption of the hovering point is still true and the inertias of the quadrotor are nearly equal (i.e., $I_{xx}≅I_{yy}≅I_{zz}$). Then, the dynamics of the ψ subsystem is represented as a double integrator in the following form:

(31) $$\{\begin{array}{l} \dot{\psi }=r\\ \dot{r}=\frac{\tau_{z}}{I_{zz}} \end{array}$$

which can be controlled using the consensus controller given in Equation (13); for simplicity, γ is assumed to equal one (γ=1), because its effect will be reflected on the value of the consensus controller gain ($K_{\psi 2}$) and adding the formation position terms. The controller of the ψ subsystem is given as

(32) $$U_{\psi }{}_{i}=\tau_{z}=\overset{N}{\underset{j=1}{\sum }}\ell_{ij}\left(K_{\psi 1}\left(\psi_{i}-\psi_{j}-\varrho_{\psi i}\right)+K_{\psi 2}\left(r_{i}-r_{j}\right)\right)$$

where $\varrho_{zi}$ and $\varrho_{\psi i}$ are the reference state in the z and ψ, respectively, and $K_{z1},\;K_{z2},\;K_{\psi 1}$ and $K_{\psi 2}\in R$ are the consensus control parameters that need to be tuned and optimized to give better performance. The overall quadrotor system with the consensus controller is shown in Figure 6.

As shown in Figure 6, the local control units (i.e., IADRC) of the z and ψ subsystems were removed because the IADRC units keep tracking reference signals, which in this case must be zero signal. Therefore, the agents’ states will be forced to reach zero. This causes canceling of the consensus control effect.

In contrast, the ϕ and θ subsystems do not have consensus controllers, while the local control units are still in place. This is due to the fact that the ϕ and θ subsystems have their own desired signals generated internally from the x and y position subsystems, as explained earlier, and can be treated as an internal part of the system. Therefore, adding consensus controllers will affect the x and y position subsystems and will never achieve the desired formation.

**Remark** **1.** The linearized subsystems in Equation (23), Equation (28) and Equation (31) of the 6-DOF quadrotor are used only for theoretical design of the controller to choose the appropriate one, while the complete nonlinear model represented by Equation (1) is used in all the simulations.

## 4. Simulation Results and Discussion

**Figure 7:**
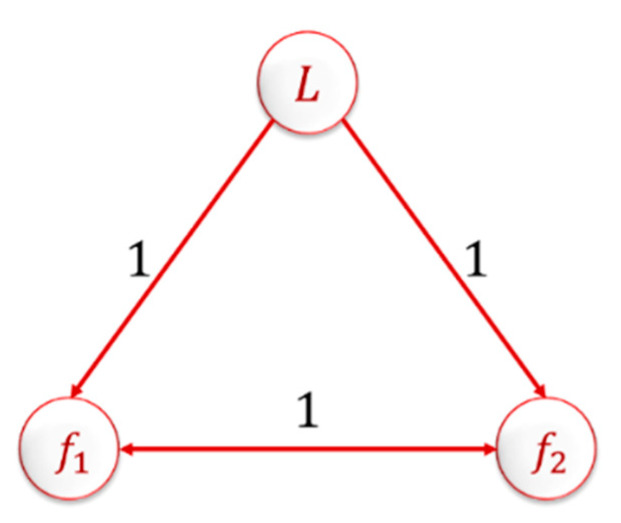
Three quadrotors leader–follower connection topology.

In this section, a MAS of three quadrotors using the leader–follower topology for communication network was studied, as shown in Figure 7. All quadrotor agents’ parameters values are defined in Table 2. The leader agent control system (NLPID–IADRC combination) parameters are given in Table 3, Table 4, Table 5 and Table 6.

The Laplacian matrix for the MAS based on the connection topology shown in Figure 7 is given as

$$L\left(D\right)=\left[\begin{array}{ccc} 0 & 0 & 0\\ -1 & 2 & -1\\ -1 & -1 & 2 \end{array}\right]$$

where the first row represents the leader agent, which does not receive any information from the followers; it just transmits its information to them. The weights used for the connection were ones. This represents that there was information transfer between the agents.

The single agent system has only one error for each state, while for the MAS the number of errors depends on the number of agents in the system. In the next simulations, for each state of the quadrotor, there were three errors (i.e., for state κ the errors were $\kappa_{1}-\kappa_{2}$, $\kappa_{1}-\kappa_{3}$, and $\kappa_{2}-\kappa_{3}$ where κ∈{x,y,z,ψ}). For the reason mentioned previously, the multi-objective performance index (OPI) used here depended on the integral time absolute error (ITAE) performance index, which is given as

(33) $$\{\begin{array}{l} opi_{i}=\overset{3}{\underset{j=1}{\sum }}\omega_{ij}\times ITAE_{ij}\\ OPI=\underset{i}{\sum }\left({\hat{\omega }}_{i}\times opi_{i}\right),\;i=x,y,z,\;\psi \end{array}$$

where $ITAE_{ij}$ represents the $ITAE=\int_{0}^{tf}\left|e\left(t\right)\right|dt$ for each of the three consensus errors. $\omega_{ij}$ was chosen to be $\frac{1}{3}$ for all i and j, while ${\hat{\omega }}_{i}$ was chosen to be 0.25 for all i.

The tuning process to obtain the optimum OPI was done using random initial values for [x,y,z,ψ] states, such as [0.001,0.001,0.001,0], [0.5,0.5,0.001,0], and [−0.5,−0.5,0.001,0] for the leader, the first follower, and the second follower, respectively. The reference signals used were zero for x, y, and ψ, while a step input was used for the altitude (z), and taking into considerations $\varrho_{xi}$, $\varrho_{yi}$, $\varrho_{zi}$, and $\varrho_{\psi i}$ mentioned in Equation (26), Equation (29) and Equation (32), respectively, all were equal to zero during the tuning process.

All the values of the consensus control parameters are shown in Table 7. The performance indices and the OPI values after GA tuning are given in Table 8 based on the initial values and the reference signals mentioned.

Some tests were done on the MAS with the proposed consensus control scheme. These are represented as follows.

### 4.1. Achieving Consensus

The purpose of this test was to check the errors between the agents’ states while following the leader agent and the time it took to reach consensus between the agents. The initial states for each agent are given in Table 9, while the reference signals applied for the altitude (z), position (x, y), and the Yaw angle (ψ) were zero signal with simulation time $t_{f}=10\;s$.

**Figure 8:**
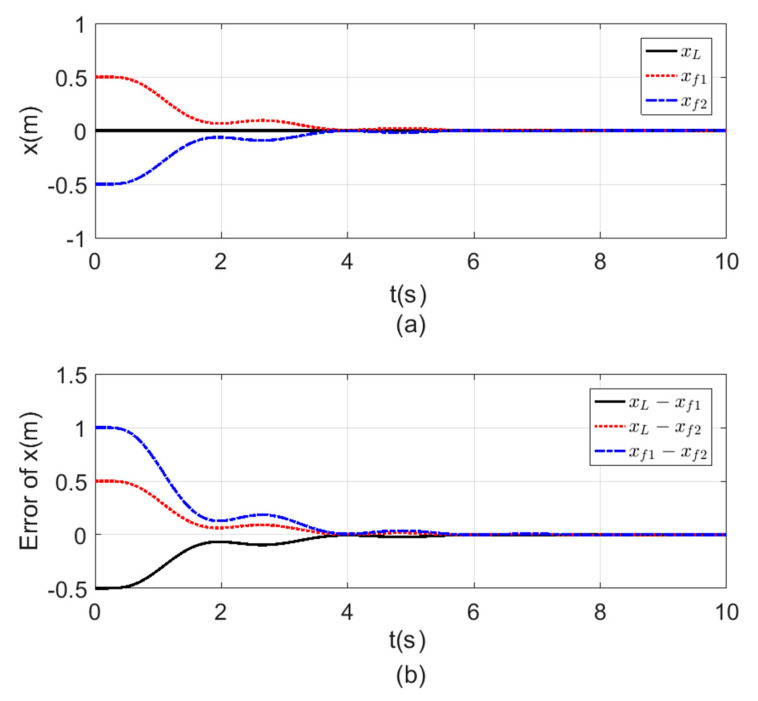
Consensus in x-position (**a**) x-position time response. (**b**) Error of the x-position.

**Figure 9:**
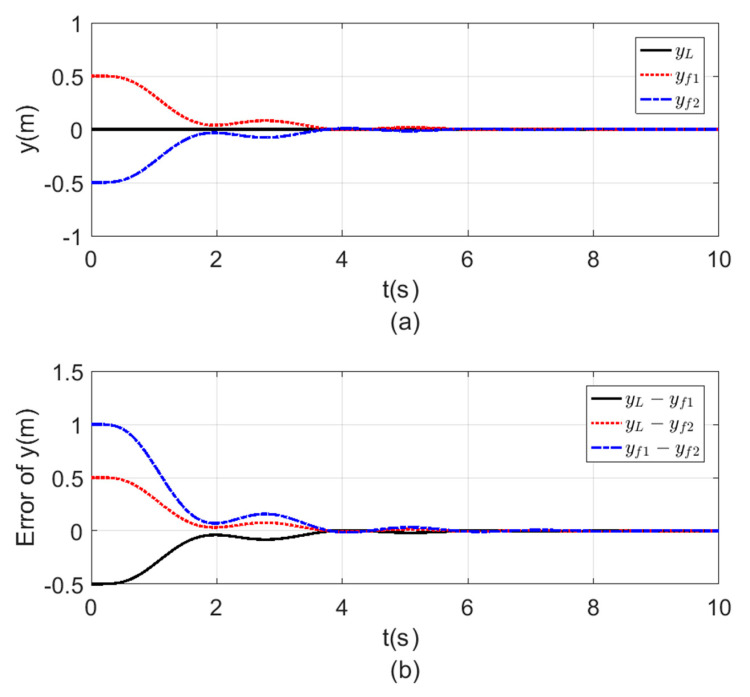
Consensus in y-position (**a**) y-position time response. (**b**) Error of the y-position.

**Figure 10:**
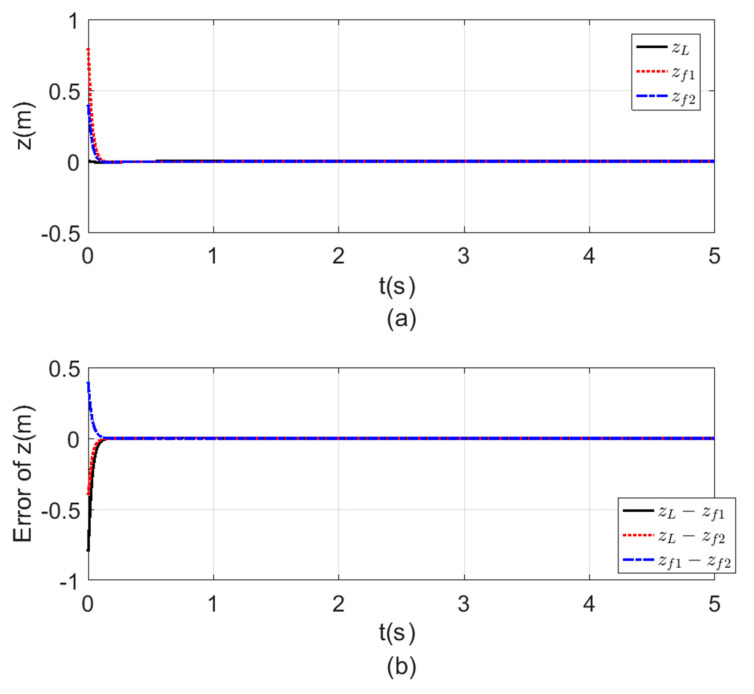
Consensus in z-position (**a**) z-position time response. (**b**) Error of the z-position.

**Figure 11:**
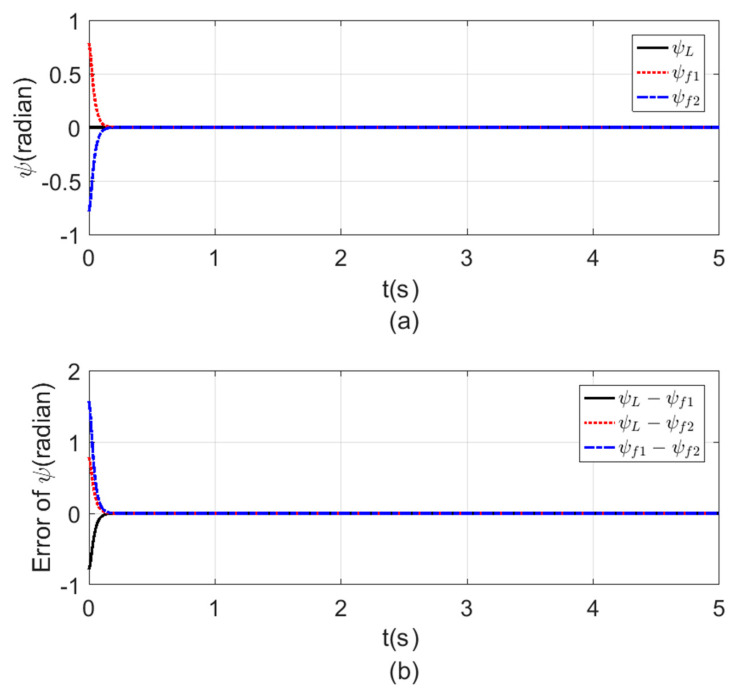
Consensus in ψ-angle (**a**) ψ-angle time response. (**b**) Error of the ψ angle.

Figure 8, Figure 9, Figure 10 and Figure 11 a show the time response for the altitude (z), position (x, y), and the Yaw angle (ψ) states, while Figure 8, Figure 9, Figure 10 and Figure 11 b show the errors between agents. The subscript L refers to the leader, while f refers to the followers. The smoothness in the time response for all cases was very clear with fast consensus-reaching (i.e., less than 4 s for the x and y position, while for the altitude z and the yaw angle ψ the time taken to reach consensus was less than 0.5 s). The errors occurring in all the states always converged to zero after a very short time even if the initial states were different.

### 4.2. Fixed Triangle Formation

A fixed formation for the agents was reached, while the leader was supposed to keep increasing in its altitude. The initial states for this test and the tests coming after were the same, as represented in Table 9, taking into consideration that the followers' initial altitude was the same altitude as that of the leader (i.e., initial z=0.001 for both followers). The reference signals of the leader for the x, y, and ψ were zero signal, while for z a ramp input was used with a slope =0.2 m with simulation time $t_{f}=30$. The values of the reference states to form the triangle shape ($\varrho_{xi}$, $\varrho_{yi}$, $\varrho_{zi}$, and $\varrho_{\psi i}$) mentioned in Equation (26), Equation (29) and Equation (32), respectively, are given as

$$\varrho_{xL}=0,\;\varrho_{xf_{1}}=1,\;\varrho_{xf_{2}}=-1;\;\varrho_{yL}=0,\;\varrho_{yf_{1}}=\varrho_{yf_{2}}=1;$$

$$\varrho_{zL}=\varrho_{zf_{1}}=\varrho_{zf_{2}}=0;\;\varrho_{\psi L}=\varrho_{\psi f_{1}}=\varrho_{\psi f_{2}}=0.$$

**Figure 12:**
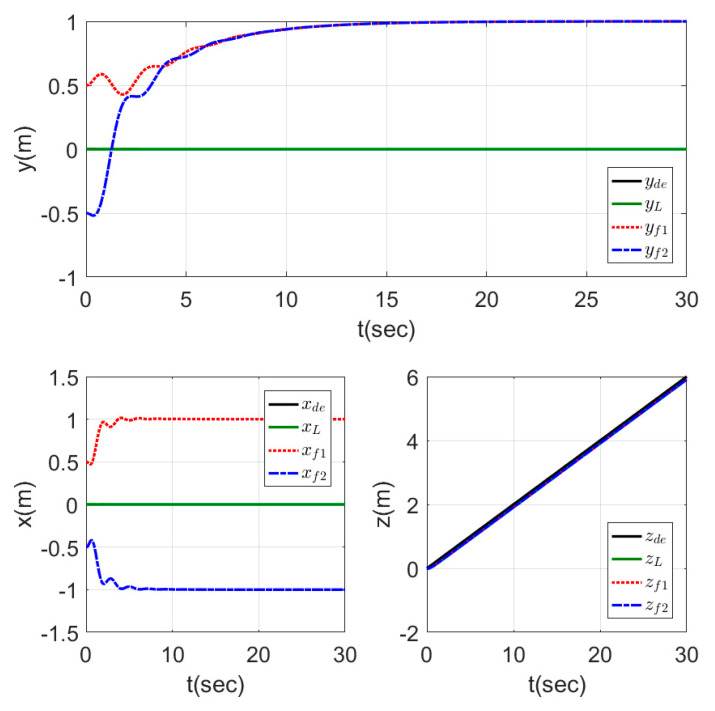
Time response of fixed triangle formation for MAS.

**Figure 13:**
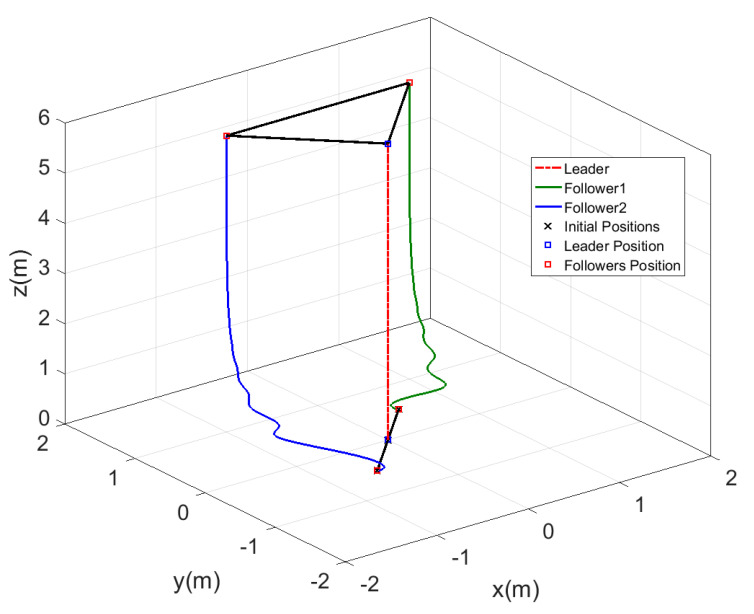
Three dimensional view of fixed triangle formation for MAS.

The time response for the x−y position and the altitude z of the leader and the two followers are shown in Figure 12, while the motion of the agents in 3D is represented in Figure 13. From the results represented in Figure 12, the followers tracked the leader trajectory perfectly with fast response, keeping the distances given previously to form the triangle shape, while the altitude of the followers matched the leader path without a variation in the response. The motion in Figure 13 of the agents shows the agents, while changing their positions to form the triangle shape, which was formed by the three agents after a few seconds from starting the test, and the shape was kept fixed while the leader altitude was increasing.

### 4.3. Fixed Formation Tracking Ramp Trajectory

This test differed from the previous one by the reference signals used with the leader to track them, where the (x and y) position was a changeable not zero signal. Forming a fixed triangle shape while the agents were moving was required in this test. The reference states values for the triangle shape are given as follows:

$$\varrho_{xL}=\varrho_{xf_{1}}=0,\;\varrho_{xf_{2}}=-1;\;\varrho_{yL}=\varrho_{yf_{2}}=0,\;\varrho_{yf_{1}}=-1;$$

$$\varrho_{zL}=\varrho_{zf_{1}}=\varrho_{zf_{2}}=0;\;\varrho_{\psi L}=\varrho_{\psi f_{1}}=\varrho_{\psi f_{2}}=0.$$

The reference trajectory signals for the leader agent were a ramp input signal for the x and y position states with starting time =10 s and slope =0.01 m, a step input for the altitude z, and a zero signal for the yaw angle ψ, with a full simulation time $t_{f}=500\;s$.

**Figure 14:**
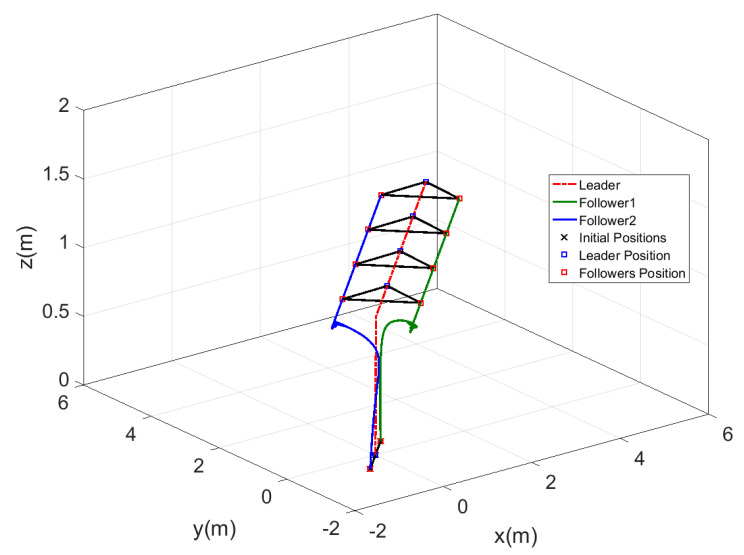
Three-dimensional view of fixed formation tracking ramp trajectory.

**Figure 15:**
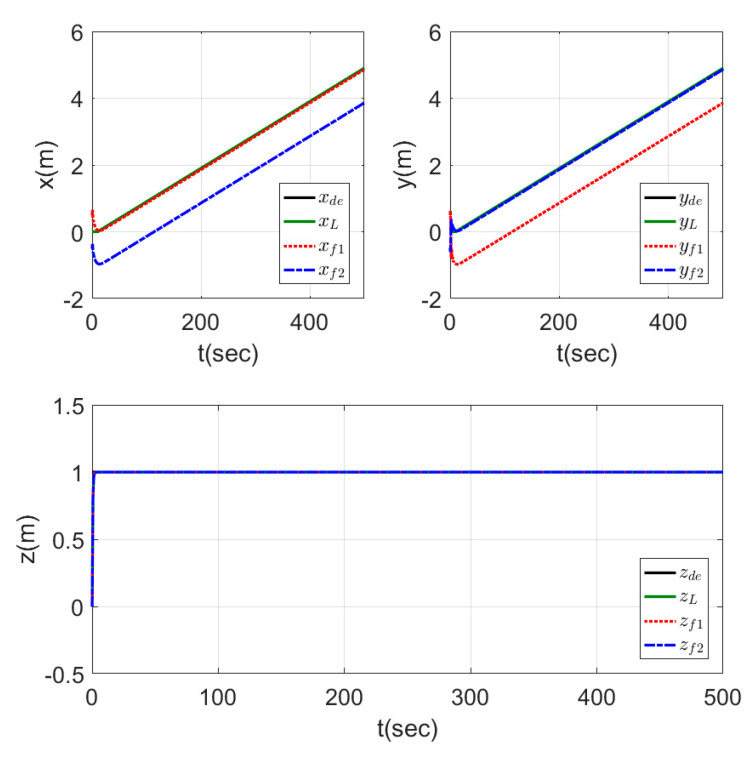
Time response of fixed formation tracking ramp trajectory.

Figure 14 shows the motion of the leader while tracking the reference trajectory and the followers keeping the required shape while tracking the leader trajectory. The shape was well performed by the three agents using the proposed controllers. The time response for this test is represented in Figure 15, which shows the fast-tracking of the followers to make the required shape with the leader agent without peaks and with a very smooth response.

### 4.4. Leader Orientation-Based Formation Tracking a Helical Trajectory

The previous test showed good trajectory tracking performance while keeping the formation shape fixed. This test differed from the previous one by following the rotation of the leader and keeping the formation shape, but rotation occurred when the leader changed its position. This test is very important for the search and rescue applications of the MAS. In this test, the helical path was used as a reference trajectory for the leader agent. These reference signals are given in Table 10.

Another difference in this test as compared to all the cases mentioned previously is that the reference states ($\varrho_{xi}$, $\varrho_{yi}$, $\varrho_{zi}$, and $\varrho_{\psi i}$) were time-varying here to guarantee that the same triangle shape was kept depending on the leader orientation. The signals of the reference states of the x and y position are given in Table 11, while the altitude and the yaw angle reference states ($\varrho_{zi}$, and $\varrho_{\psi i}$) were zero for all agents.

**Figure 16:**
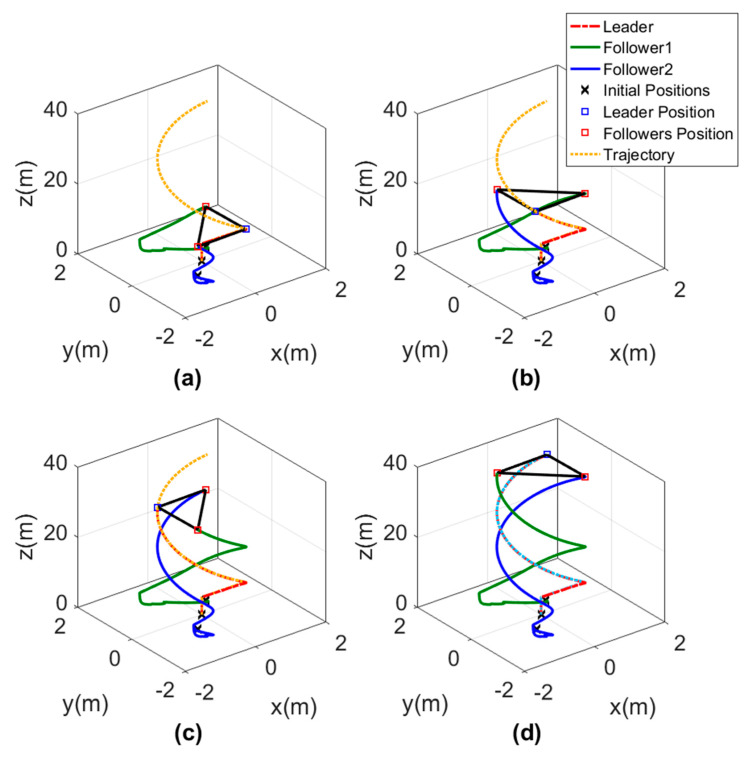
Three-dimensional views of leader orientation-based formation tracking a helical trajectory, (**a**) Agents position at t=50 s. (**b**) Agents position at t=100 s. (**c**) Agents position at t=150 s. (**d**) Agents position at t=200 s.

The 3D motion of the agents while forming the triangle shape is shown in Figure 16. The triangle shape was well performed by the agents depending on the orientation of the leader and the path it was following. In Figure 16, each subgraph represents the triangle shape formed by the agents at a different time to show that the orientation of the triangle was changing depending on the leader direction.

**Figure 17:**
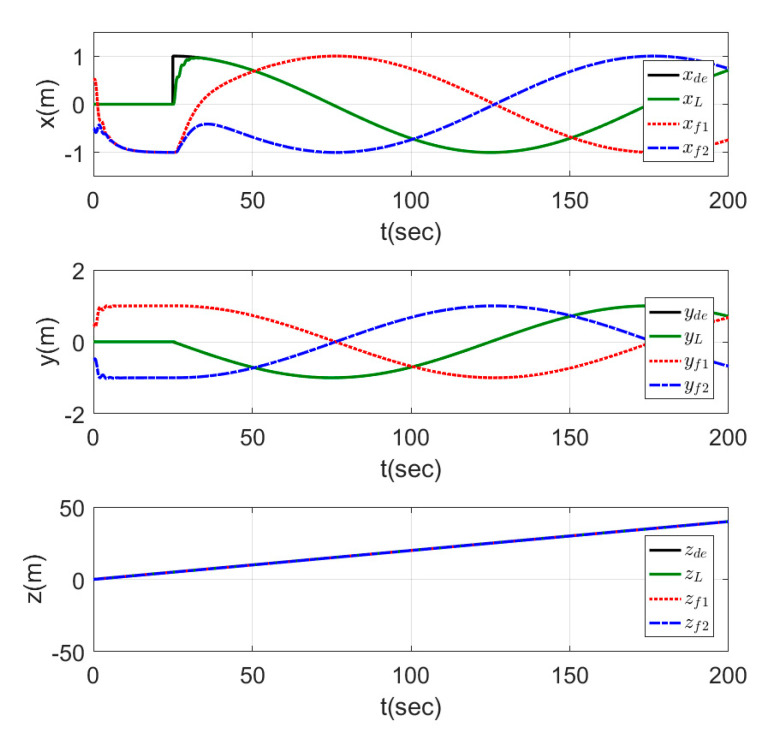
Time response of leader orientation-based formation tracking a helical trajectory.

The time responses are presented in Figure 17 for the x, y, and z states. The results show a good tracking for the desired signals, while the leader direction was changing the value of the reference states ($\varrho_{x}$ and $\varrho_{y}$) for the two follower agents (i.e., in second 75 of the simulation $\varrho_{xf_{1}}=1$, $\varrho_{xf_{2}}=-1$, and $\varrho_{yf_{1}}=\varrho_{yf_{2}}=1$, which represented a triangle shape directed into the −ve (y) direction, while in second 125 of the simulation $\varrho_{xf_{1}}=\varrho_{xf_{2}}=1$, and $\varrho_{yf_{1}}=-1$, $\varrho_{yf_{2}}=1$, which represented a triangle shape with the same dimensions but directed into the –ve (x) direction). The mentioned two examples from the results showed that the shape was kept, while its direction was changed depending on the leader agent.

### 4.5. Switching Formation Topology

This test required changing the formation shape during hovering mode for the MAS. The reference trajectory signals used for the leader agent are given in Table 12, while the state references for x and y used are given in Table 13.

**Figure 18:**
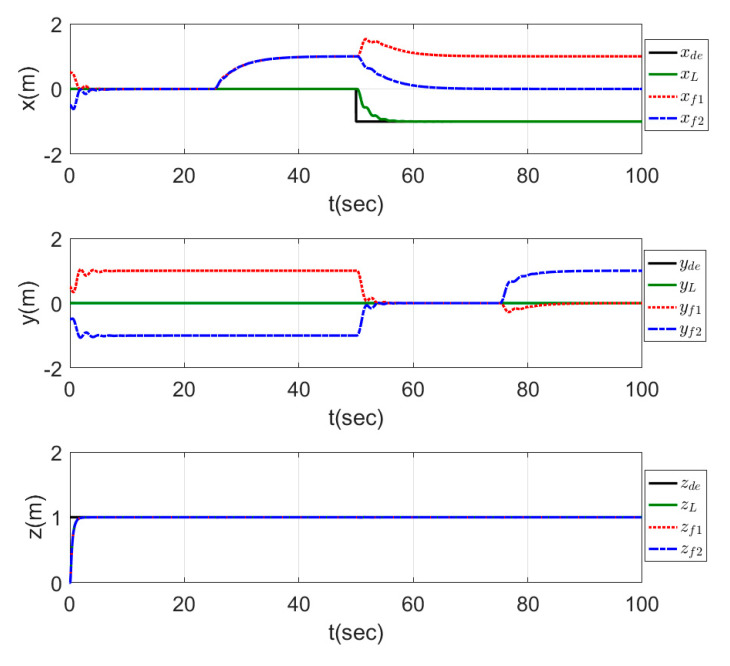
Time response of switching formation topology.

The results of this test are represented in the plots of Figure 18, which show that the agents formed a line shape on the y-direction in a fast response time through the interval (0−25) s of the time. In the second time interval (25−50) s, a triangle shape headed by the leader agent and directed to the –ve x-direction was performed by the agents in a smooth and fast response. In the third time interval (50−75) s, the leader started to move to the –ve y-direction, while the agents formed a line shape on the x-direction, and the shape was well performed. In the last time interval (75−100) s, another triangle shape was performed but this time was headed by the second follower and was directed to the +ve y-direction. 

**Figure 19:**
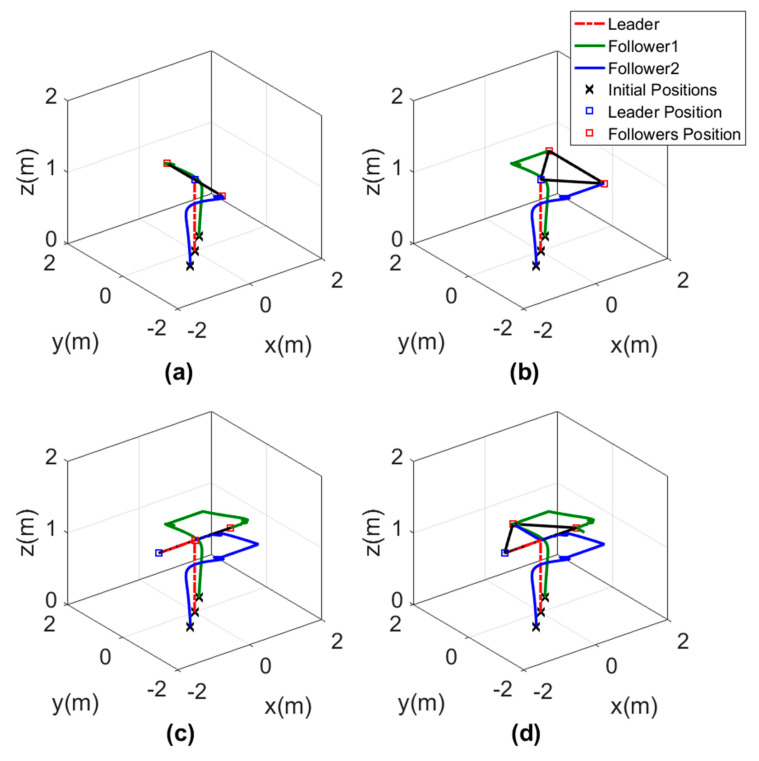
Three-dimensional views of switching formation topology, (**a**) Agents formation at t=25 s. (**b**) Agents formation at t=50 s. (**c**) Agents formation at t=75 s. (**d**) Agents formation at t=100 s.

In this test, the 3D motion in Figure 19 was represented in multiple subgraphs to show the exact formation achieved by the MAS. These subgraphs present the motion sequence of the three agents, and it is clear that all the desired formations were performed perfectly without any error.

### 4.6. Fixed Formation Tracking with Exogenous Disturbances

In this test, the same trajectory and parameters of the fixed formation tracking ramp trajectory test (third test) were used in addition to the existence of different disturbances applied on the leader agent to show the ability of the proposed controller to cope with such practical case. The disturbances applied on the different states of the leader agent were a step of $f_{wz}=100N$ at t=50 s, pulses for the roll, and pitch and yaw angles ($\tau_{wx}=1N\cdot m$ at t=150 s, $\tau_{wy}=1N\cdot m$ at t=250 s, $\tau_{wz}=1N\cdot m$ at t=300 s) with pulse widths of 50 s.

**Figure 20:**
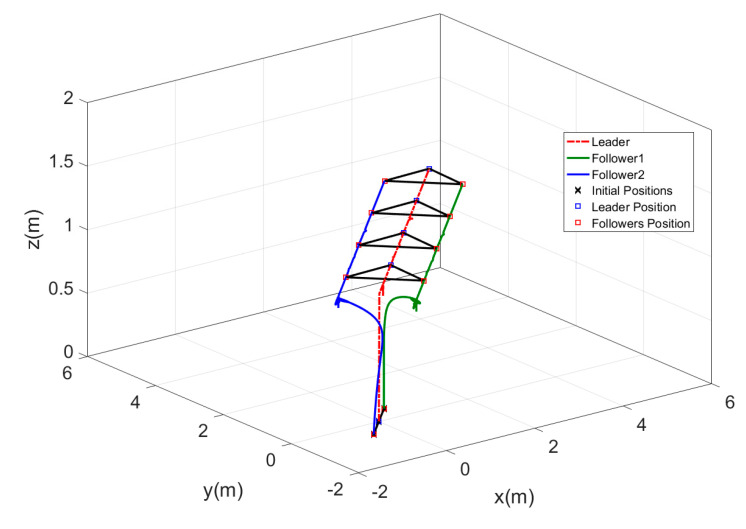
Three-dimensional view of fixed formation tracking with disturbances.

**Figure 21:**
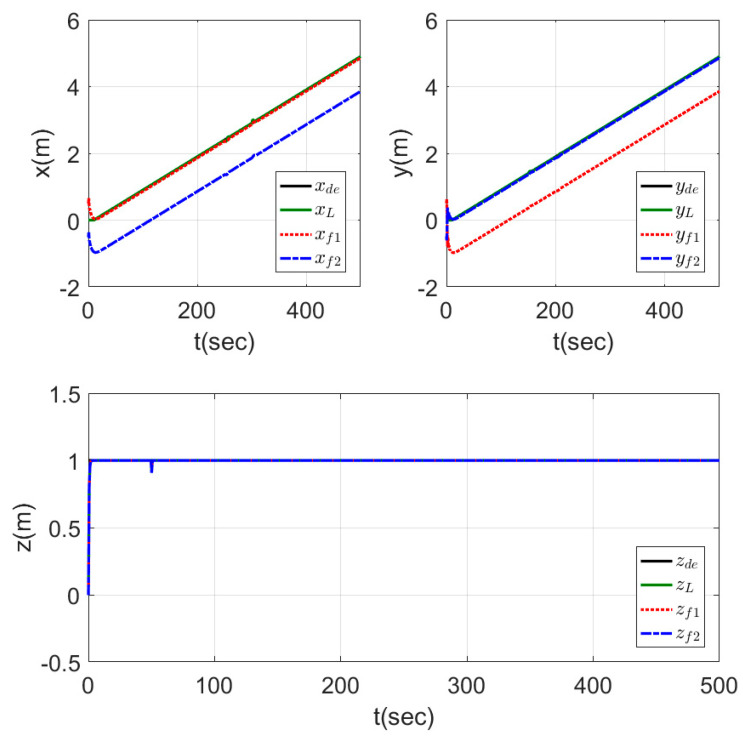
Time response of fixed formation tracking with disturbances.

Figure 20 and Figure 21 show the 3D motion of the agents and the time response of each agent while tracking the trajectory and the disturbances applied. The agents performed the trajectory tracking while different disturbances existed in a perfect way, where small peaks and fast tracking are shown in Figure 21. This proves the high immunity of the proposed controlling scheme against external disturbances.

## 5. Conclusions and Future Aspects

The consensus control proposed for three agents of quadrotors in this work showed good performance in time. From the simulation cases and the results presented, it can be concluded that the complex nonlinear model for the quadrotor system is treated as a multiple double integrator subsystem. As compared to the other simplified quadrotor models used by some researchers mentioned in the literature survey, the proposed controller outperformed the others by dealing with the complete nonlinear model of the quadrotor system with good performance. This work can be further developed by taking into consideration the following points: proving the closed-loop stability of the proposed control scheme with the nonlinear quadrotor model, developing the proposed controller to achieve containment and flocking control, and implementing the consensus control on a hardware platform using the proposed controlling scheme. Another direction is to extend other control design methods, as in [29,30], to control the single agent UA based on its nonlinear model while using the proposed consensus control scheme.

## Figures and Tables

**Table 1:** Parameter descriptions.

Parameters	Description	Units
[x y z]	Linear position vector	m
[ϕ θ ψ]	Angular position vector	Rad
[u v w]	Linear velocity vector	m/s
[p q r]	Angular velocity vector	Rad/s
$\left[I_{xx}\;I_{yy}\;I_{zz}\right]$	Moment of inertia vector	kg·m^2^
$f_{t}$	Total thrust generated by rotors	N
$\left[\tau_{x}\;\tau_{y}\;\tau_{z}\right]$	Control torques	N m
$\left[f_{wx}\;f_{wy}\;f_{wz}\right]$	Wind force vector	N
$\left[\tau_{wx}\;\tau_{wy}\;\tau_{wz}\right]$	Wind torque vector	N m
g	Gravitational force	m/s^2^
m	Total mass	kg
c( )≡cos( ), s( )≡sin( ), and t( )≡tan( )		

**Table 2:** Quadrotor model parameters.

Parameter	Value	Description	Unit
$I_{xx}$	$8.5532\times 10^{-3}$	Moment of inertia around x-axis	$\mathrm{kg}\cdot m^{2}$
$I_{yy}$	$8.5532\times 10^{-3}$	Moment of inertia around y-axis	$\mathrm{kg}\cdot m^{2}$
$I_{zz}$	$1.476\times 10^{-2}$	Moment of inertia around z-axis	$\mathrm{kg}\cdot m^{2}$
g	$9.81\times 10^{0}$	Gravitational force	$m/s^{2}$
m	$9.64\times 10^{-1}$	Total mass	kg
b	$7.66\times 10^{-5}$	Thrust coefficient	$N\cdot s^{2}$
d	$5.63\times 10^{-6}$	Drag coefficient	$N\cdot m\cdot s^{2}$
l	$2.2\times 10^{-1}$	Motor to center length	m

**Table 3:** LESOs Parameters of the leader quadrotor.

	z	ϕ	θ	ψ
$\omega_{0}$	300	861.36	671.76	749.05
$b_{0}$	0.5	0.0041	0.005	0.0047

**Table 4:** ITD parameters of the leader quadrotor.

	z, ϕ, θ, ψ
α	0.9789
β	2.7936
γ	16.7728
R	26.5005

**Table 5:** NLPD parameters of the leader quadrotor.

	z	ϕ	θ	ψ
$k_{11}$	32.4800	5.6390	0.51080	0.6993
$k_{12}$	11.4360	0.0764	0.0390	0.2109
$k_{21}$	9.0756	0.7495	0.0660	0.2415
$k_{22}$	0.1518	0.0471	0.0666	0.1024
$\mu_{1}$	0.2814	0.0763	0.5191	0.1272
$\mu_{2}$	0.4234	0.5997	0.7747	0.3760
$\alpha_{1}$	0.9684	0.9591	0.9574	0.9741
$\alpha_{2}$	0.9580	0.9547	1.0034	0.9417

**Table 6:** NLPID parameters for the x−y position of the leader quadrotor.

	x	y		x	y
$k_{11}$	3.0735	2.3046	$\mu_{1}$	0.1214	0.1057
$k_{12}$	0.5674	0.1108	$\mu_{2}$	0.9859	0.9744
$k_{21}$	6.0204	4.5269	$\mu_{3}$	0.7656	0.6008
$k_{22}$	2.0076	2.0040	$\alpha_{1}$	0.9106	0.9101
$k_{31}$	$2.2409\times 10^{-4}$	$2.3828\times 10^{-4}$	$\alpha_{2}$	0.9999	0.9994
$k_{32}$	$5.5135\times 10^{-7}$	$6.8046\times 10^{-7}$	$\alpha_{3}$	0.9140	0.9137

**Table 7:** Consensus controller parameters.

x		y	
$k_{x}$	$K_{x}$	$k_{y}$	$K_{y}$
4.0259	0.9798	3.9732	1.0631
z		ψ	
$K_{z1}$	$K_{z1}$	$K_{\psi 1}$	$K_{\psi 2}$
14,169.9808	427.2904	13.8067	0.5709

**Table 8:** Performance indices and OPI values for consensus control.

	Consensus Control		
	ITAE1	ITAE2	ITAE3
x	1.076761	1.009015	1.879853
y	1.203705	1.177282	2.106002
z	0.838854	0.838702	0.001418
ψ	0.005238	0.005962	0.001104
OPI	0.845325		

**Table 9:** Achieving consensus test initial states.

	Initial States			
	x	y	z	ψ
Leader	0.001	0.001	0.001	0
Follower 1	0.5	0.5	0.8	π/4
Follower 2	−0.5	−0.5	0.4	−π/4

**Table 10:** Leader orientation-based formation tracking a helical trajectory input signals.

State	Reference Trajectory	Time (s)
x	cos(0.01πt)	$[25,t_{f}$ *]
y	sin(0.01πt)	$[25,t_{f}$ *]
z	0.2 t	$[0,t_{f}$ *]
ψ	0	$[0,t_{f}$ *]

* $t_{f}=200\;s$.

**Table 11:** The x and y reference states.

	Leader	Follower 1	Follower 2
$\varrho_{x}$	0	$-\cos\left(\theta_{L}\right)+\sin\left(\theta_{L}\right)$	$-\cos\left(\theta_{L}\right)+\sin\left(\theta_{L}\right)$
$\varrho_{y}$	0	$\cos\left(\theta_{L}\right)-\sin\left(\theta_{L}\right)$	$-\cos\left(\theta_{L}\right)-\sin\left(\theta_{L}\right)$
$\theta_{L}=\tan^{-1}\frac{y_{L}}{x_{L}}$			

**Table 12:** Switching formation topology input signals.

State	Reference Trajectory	Time (s)
x	−u(t−50)	$[110,t_{f}$ *]
z	u(t)	$[0,t_{f}$ *]
y, ψ	0	$[0,t_{f}$ *]

* $t_{f}=100\;s$.

**Table 13:** The x and y reference states for changing formation.

	Leader	Follower 1	Follower 2
$\varrho_{x}$	0	u(t−25)+u(t−50)	u(t−25)
$\varrho_{y}$	0	1−u(t−50)	−1+u(t−50)+u(t−75)

**Figure A1:**
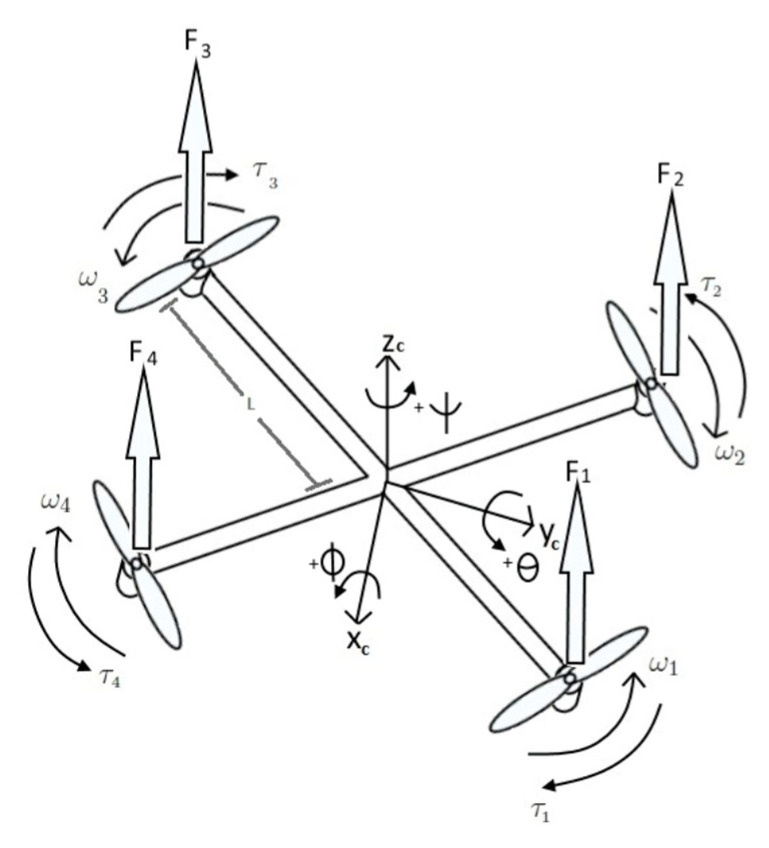
Quadrotor coordinate systems.

**Figure A2:**
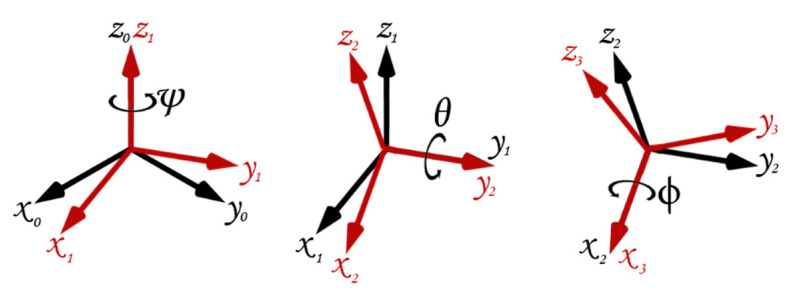
Rotation around Z, Y, and X representation.

## Appendix A

### Appendix A.1. Coordinate Systems

There are two reference frames for the quadrotor system, which are called inertial frame (I) and body frame (K). The inertial frame is a fixed frame (e.g., ground), which means it is fixed relative to the body frame, while the body frame is fixed to the quadrotor system, and it depends on the quadrotor motion [31]. Figure A1 illustrates the two coordinate systems.

### Appendix A.2. Euler Angles

Leonhard Euler describes the angular position for a rigid body by using three angles (roll “ϕ”, pitch “θ”, and yaw “ψ”), which are called (Euler angles). These three angles can be given in different ways to describe the angular position in 3D Euclidean space. Euler angles can be used to specify the orientation of a frame relative to another frame, which can be obtained by three successive rotations [31]. In this model, ZYX rotation (ψ−θ−ϕ) was used, as shown in Figure A2, and described by the following matrices:

(A1) $$R_{x}\left(\phi \right)=\left[\begin{array}{ccc} 1 & 0 & 0\\ 0 & c\left(\phi \right) & -s\left(\phi \right)\\ 0 & s\left(\phi \right) & c\left(\phi \right) \end{array}\right]$$

(A2) $$R_{y}\left(\theta \right)=\left[\begin{array}{ccc} c\left(\theta \right) & 0 & s\left(\theta \right)\\ 0 & 1 & 0\\ -s\left(\theta \right) & 0 & c\left(\theta \right) \end{array}\right]$$

(A3) $$R_{z}\left(\psi \right)=\left[\begin{array}{ccc} c\left(\psi \right) & -s\left(\psi \right) & 0\\ s\left(\psi \right) & c\left(\psi \right) & 0\\ 0 & 0 & 1 \end{array}\right]$$

(A4) $$\begin{array}{l} R_{zyx}\left(\phi ,\theta ,\psi \right)=R_{z}\left(\psi \right).R_{y}\left(\theta \right).R_{x}\left(\phi \right)\\ =\left[\begin{array}{ccc} c\left(\theta \right)c\left(\psi \right) & s\left(\phi \right)s\left(\theta \right)c\left(\psi \right)-c\left(\phi \right)s\left(\psi \right) & c\left(\phi \right)s\left(\theta \right)c\left(\psi \right)+s\left(\phi \right)s\left(\psi \right)\\ c\left(\theta \right)s\left(\psi \right) & s\left(\phi \right)s\left(\theta \right)s\left(\psi \right)+c\left(\phi \right)c\left(\psi \right) & c\left(\phi \right)s\left(\theta \right)s\left(\psi \right)-s\left(\phi \right)c\left(\psi \right)\\ -s\left(\theta \right) & s\left(\phi \right)c\left(\theta \right) & c\left(\phi \right)c\left(\theta \right) \end{array}\right] \end{array}$$

where c( )=cos( ), and s( )=sin( ).

### Appendix A.3. Model Assumptions

To control a nonlinear, complex, and severely under-actuated system such as a quadrotor system, some assumptions need to be made during the quadrotor modeling process [31]:

- The quadrotor is rigid.
- The quadrotor structure is symmetric.
- The ground effect is neglected.
- The center of gravity origin and the principal axes coincide with the body frame origin and axes.
- Input forces and torques proportional to the squared angular velocity of the rotors.

### Appendix A.4. Mathematical Model

The quadrotor nonlinear mathematical model is based on Newton and Euler equations. The two frames (i.e., inertial frame and body frame) are related to each other by the following relations:

(A5) $$\{\begin{array}{l} \overset{}{V}=R\cdot V_{B}\\ \dot{W}=T\cdot W_{B} \end{array}$$

where $V={\left[x\;y\;z\right]}^{T}\in R^{3}$ is the linear position vector in the earth frame, $W={\left[\phi \;\theta \;\psi \right]}^{T}\in R^{3}$ is the angular position vector in the earth frame, $V_{B}={\left[u\;v\;w\right]}^{T}\in R^{3}$ is the linear velocity vector in the body frame, $W_{B}={\left[p\;q\;r\right]}^{T}\in R^{3}$ is the angular velocity vector in the body frame, $R=R_{zyx}$, and T is the angular transformation matrix, which is given by [31]

(A6) $$T=\left[\begin{array}{ccc} 1 & s\left(\phi \right)t\left(\theta \right) & c\left(\phi \right)t\left(\theta \right)\\ 0 & c\left(\phi \right) & -s\left(\phi \right)\\ 0 & \frac{s\left(\phi \right)}{c\left(\theta \right)} & \frac{c\left(\phi \right)}{c\left(\theta \right)} \end{array}\right]$$

where t( )=tan( ). The quadrotor kinematic model is given as

(A7) $$\{\begin{array}{l} \dot{x}=c\left(\psi \right)c\left(\theta \right)u+\left[c\left(\psi \right)s\left(\phi \right)s\left(\theta \right)-c\left(\phi \right)s\left(\psi \right)\right]v+\left[s\left(\phi \right)s\left(\psi \right)+c\left(\phi \right)c\left(\psi \right)s\left(\theta \right)\right]w\\ \dot{y}=c\left(\theta \right)s\left(\psi \right)u+\left[c\left(\phi \right)c\left(\psi \right)+s\left(\phi \right)s\left(\psi \right)s\left(\theta \right)\right]v+\left[c\left(\phi \right)s\left(\psi \right)s\left(\theta \right)-c\left(\psi \right)s\left(\phi \right)\right]w\\ \dot{z}=-s\left(\theta \right)u+c\left(\theta \right)s\left(\phi \right)v+c\left(\phi \right)c\left(\theta \right)w\\ \dot{\phi }=p+s\left(\phi \right)t\left(\theta \right)q+c\left(\phi \right)t\left(\theta \right)r\\ \dot{\theta }=c\left(\phi \right)q-s\left(\phi \right)r\\ \dot{\psi }=\frac{s\left(\phi \right)}{c\left(\theta \right)}q+\frac{c\left(\phi \right)}{c\left(\theta \right)}r \end{array}$$

The total force applied on the quadrotor system is stated by Newton’s law, while the total torque acting on the quadrotor is given by Euler’s equation. Both total force and total torque are given by the following relations:

(A8) $$\{\begin{array}{l} m\left(W_{B}\times V_{B}+{\dot{V}}_{B}\right)=f_{B}\\ I.{\dot{W}}_{B}+W_{B}\times \left(I.W_{B}\right)=m_{B} \end{array}$$

where m is the quadrotor mass, × is the cross product sign, $f_{B}={\left[f_{x}\;f_{y}\;f_{z}\right]}^{T}\in R^{3}$ is the total force, $m_{B}={\left[m_{x}\;m_{y}\;m_{z}\right]}^{T}\in R^{3}$ is the total torque, and I is the inertia matrix, which is given as [31]

$$I=\left[\begin{array}{ccc} I_{xx} & 0 & 0\\ 0 & I_{yy} & 0\\ 0 & 0 & I_{zz} \end{array}\right]\in R^{3\times 3}$$

Then, the dynamical model for the quadrotor is given as [31]

(A9) $$\left\{ \begin{array}{l} f_{x}=m\left(\dot{u}+qw-rv\right)\\ f_{y}=m\left(\dot{v}-pw-ru\right)\\ f_{z}=m\left(\dot{w}+pv-qu\right)\\ m_{x}=\dot{p}I_{xx}-qrI_{yy}+qrI_{zz}\\ m_{y}=\dot{q}I_{yy}+prI_{xx}-prI_{zz}\\ m_{z}=\dot{r}I_{zz}-pqI_{xx}+pqI_{yy} \end{array}\right.$$

### Appendix A.5. Quadrotor Forces and Moments

Several external forces are applied to the quadrotor and are given here:

(A10) $$f_{B}=mgR^{T}\cdot {\hat{k}}_{I}-f_{t}{\hat{k}}_{B}+f_{w}$$

where ${\hat{k}}_{I}$ is the inertial z-axis unit vector, ${\hat{k}}_{B}$ is the body z-axis unit vector, g is the gravitational acceleration, $f_{t}$ is the rotors total thrust, and $f_{w}={\left[f_{wx}\;f_{wy}\;f_{wz}\right]}^{T}\in R^{3}$ are the wind forces applied on the quadrotor system. The external moments applied on the quadrotor system are given as

(A11) $$m_{B}=\tau_{B}+\tau_{w}-m_{g}$$

where $\tau_{B}={\left[\tau_{x}\;\tau_{y}\;\tau_{z}\right]}^{T}\in R^{3}$ are the rotor speeds differences torques, $\tau_{w}={\left[\tau_{wx}\;\tau_{wy}\;\tau_{wz}\right]}^{T}\in R^{3}$ are the wind torques applied on the quadrotor system, and $m_{g}$ is the gyroscopic moments generated by a combination between the four rotors rotation and the quadrotor body, which can be described by the following relation:

(A12) $$m_{g}=\overset{4}{\underset{i=1}{\sum }}J_{p}\left(W_{B}\wedge {\hat{k}}_{B}\right){\left(-1\right)}^{i+1}\Omega_{i}$$

where $J_{p}$ is the rotor inertia, which is found to be small enough to be neglected; thus, the gyroscopic moments are excluded from the total equations. $\Omega_{i}$ is the $i^{th}$ rotor angular speed, while the ${\left(-1\right)}^{i+1}$ term represent the direction of rotation for each rotor. Then, the forces and moments are substituted in Equation (A9) to obtain the final dynamical model as follows [31]:

(A13) $$\left\{ \begin{array}{l} -mg\left[s\left(\theta \right)\right]+f_{wx}=m\left(\dot{u}+qw-rv\right)\\ mg\left[c\left(\theta \right)s\left(\phi \right)\right]+f_{wy}=m\left(\dot{v}-pw-ru\right)\\ mg\left[c\left(\theta \right)c\left(\phi \right)\right]+f_{wz}=m\left(\dot{w}+pv-qu\right)\\ \tau_{x}+\tau_{wx}=\dot{p}I_{xx}-qrI_{yy}+qrI_{zz}\\ \tau_{y}+\tau_{wy}=\dot{q}I_{yy}+prI_{xx}-prI_{zz}\\ \tau_{z}+\tau_{wz}=\dot{r}I_{zz}-pqI_{xx}+pqI_{yy} \end{array}\right.$$

### Appendix A.6. Rotor Dynamics Modeling

The inputs for the 6-DOF quadrotor system are combinations of the rotor speed (Ω), which in this case is a force $f_{t}$ to control the vertical thrust (z) and the torques ($\tau_{x}$,$\;\tau_{y}$, and $\tau_{z}$) to control the angles (ϕ, θ, and ψ), respectively. Their mathematical relations are given as [31]

(A14) $$\{\begin{array}{l} f_{t}=b\left(\Omega_{1}^{2}+\Omega_{2}^{2}+\Omega_{3}^{2}+\Omega_{4}^{2}\right)\;\\ \tau_{x}=bl\left(\Omega_{3}^{2}-\Omega_{1}^{2}\right)\\ \tau_{y}=bl\left(\Omega_{4}^{2}-\Omega_{2}^{2}\right)\\ \tau_{z}=d\left(\Omega_{2}^{2}+\Omega_{4}^{2}-\Omega_{1}^{2}-\Omega_{3}^{2}\right) \end{array}$$

where b is the thrust coefficient, l is the distance from the quadrotor center to the rotor, and d is the drag coefficient.

The overall nonlinear mathematical model for the quadrotor can be represented as

(A15) $$\{\begin{array}{ll} \left\{ \begin{array}{l} \dot{x}=c\left(\psi \right)c\left(\theta \right)u+\left[c\left(\psi \right)s\left(\phi \right)s\left(\theta \right)-c\left(\phi \right)s\left(\psi \right)\right]v+\left[s\left(\phi \right)s\left(\psi \right)+c\left(\phi \right)c\left(\psi \right)s\left(\theta \right)\right]w\\ \dot{u}=rv-qw-gs\left(\theta \right)+\frac{f_{wx}}{m} \end{array}\right. & \left(a\right)\\ \left\{ \begin{array}{l} \dot{y}=c\left(\theta \right)s\left(\psi \right)u+\left[c\left(\phi \right)c\left(\psi \right)+s\left(\phi \right)s\left(\psi \right)s\left(\theta \right)\right]v+\left[c\left(\phi \right)s\left(\psi \right)s\left(\theta \right)-c\left(\psi \right)s\left(\phi \right)\right]w\\ \dot{v}=-ru+pw+gs\left(\phi \right)c\left(\theta \right)+\frac{f_{wy}}{m} \end{array}\right. & \left(b\right)\\ \left\{ \begin{array}{l} \dot{z}=-s\left(\theta \right)u+c\left(\theta \right)s\left(\phi \right)v+c\left(\phi \right)c\left(\theta \right)w\\ \dot{w}=qu-pv+gc\left(\theta \right)c\left(\phi \right)+\frac{f_{wz}-f_{t}}{m} \end{array}\right. & \left(c\right)\\ \left\{ \begin{array}{l} \dot{\phi }=p+s\left(\phi \right)t\left(\theta \right)q+c\left(\phi \right)t\left(\theta \right)r\\ \dot{p}=\frac{I_{yy}-I_{zz}}{I_{xx}}rq+\frac{\tau_{x}+\tau_{wx}}{I_{xx}} \end{array}\right. & \left(d\right)\\ \left\{ \begin{array}{l} \dot{\theta }=c\left(\phi \right)q-s\left(\phi \right)r\\ \dot{q}=\frac{I_{zz}-I_{xx}}{I_{yy}}pr+\frac{\tau_{y}+\tau_{wy}}{I_{yy}} \end{array}\right. & \left(e\right)\\ \left\{ \begin{array}{l} \dot{\psi }=\frac{s\left(\phi \right)}{c\left(\theta \right)}q+\frac{c\left(\phi \right)}{c\left(\theta \right)}r\\ \dot{r}=\frac{I_{xx}-I_{yy}}{I_{zz}}pq+\frac{\tau_{z}+\tau_{wz}}{I_{zz}} \end{array}\right. & \left(f\right) \end{array}$$

There is another dynamic model for the quadrotor system, which is helpful in the controller design. Using Newton’s law, the following is obtained:

(A16) $$m\dot{V}=R\cdot f_{B}=mg{\hat{k}}_{I}-f_{t}R\cdot {\hat{k}}_{B}$$

(A17) $$\{\begin{array}{l} \ddot{x}=-\frac{f_{t}}{m}\left[s\left(\phi \right)s\left(\psi \right)+c\left(\phi \right)c\left(\psi \right)s\left(\theta \right)\right]\\ \ddot{y}=-\frac{f_{t}}{m}\left[c\left(\phi \right)s\left(\psi \right)s\left(\theta \right)-c\left(\psi \right)s\left(\phi \right)\right]\\ \ddot{z}=-\frac{f_{t}}{m}\left[c\left(\phi \right)c\left(\theta \right)\right]+g \end{array}$$
